# Comparison Between Early and Late Tracheostomy in ICU Patients Including COVID-19 and Non-COVID-19 Patients: A Retrospective Cohort Study at a Tertiary Care Hospital

**DOI:** 10.7759/cureus.64481

**Published:** 2024-07-13

**Authors:** Yasir Al Balushi, Jyoti Burad

**Affiliations:** 1 Anesthesia and Intensive Care, Sultan Qaboos University Hospital, Muscat, OMN

**Keywords:** ventilator, tracheostomy, mortality, length of stay, covid-19

## Abstract

Background

Tracheostomy is a common intervention for intensive care unit (ICU) patients for various reasons. The superiority of early versus late tracheostomy is still unfounded for non-COVID-19 cases. The COVID-19 pandemic complicated the matter, as little literature was available on the ideal timing of tracheostomy for patients with COVID-19.

Research question

This study aimed to establish the superiority of early or late tracheostomy for COVID-19 and non-COVID-19 cases by comparing outcomes, including ICU mortality, ventilation days after tracheostomy, and ICU length of stay (LOS).

Study design and methods

A single-center retrospective cohort study was conducted on ventilated ICU patients both with and without COVID-19 at a university hospital between January 2020 and December 2021. During the study period, 1,393 ventilated patients were scanned, and 156 were found to be tracheostomized. Tracheostomy was considered to be early when performed within 10 days of intubation, after which it was considered to be late.

Results

Tracheostomy was performed early for 84/156 (53.8%) of tracheostomized patients and late for 72/156 (46.2%) of patients. The overall mortality was 42.9% (36/84) versus 69.4% (50/72) (P=0.001, OR=3.03, 95% CI=1.563-5.874), 31.4% versus 65.5% in the non-COVID-19 group and 60.6% versus 72.1% (P=0.005, OR=2.640, 95% CI=1.345-5.181) in the COVID-19 group for the early and late tracheostomy groups, respectively. Ventilation days were higher for the late tracheostomy group than for the early tracheostomy group in the non-COVID-19 group (17.10 versus 9.18 days, P<0.001). However, it was almost the same for the early and late tracheostomy groups in the COVID-19 group (14.15 versus 13.86 days, P=0.821). The ICU LOS was greater for the late tracheostomy group for both the COVID-19 and non-COVID-19 groups. Multivariate analysis revealed that ICU mortality is significantly associated with age, ventilation days, and ICU LOS.

Interpretation

The results of this study indicate that early tracheostomy was associated with lower mortality, fewer ventilation days, and shorter LOS in both the COVID-19 and non-COVID-19 groups.

## Introduction

Tracheostomy is a simple and commonly performed bedside procedure for critically ill people who require long-term mechanical ventilation, have a low Glasgow Coma Scale, and require chest toileting. Tracheostomy reduces the complications of endotracheal intubation, such as ventilator-associated pneumonia, as well as the requirement for sedation, the number of ventilator days, and the intensive care unit (ICU) length of stay (LOS) [1].

Early tracheostomy might reduce the ICU LOS, whereas delaying the tracheostomy might avoid a few tracheostomies. A review of recent studies revealed a lower mortality rate in early tracheostomized patients than in late tracheostomized patients. A study comparing early and late tracheostomy indicated that out of eight randomized clinical trials (RCTs), seven had lower mortality rates for early tracheostomy than for late tracheostomy [2]. On the other hand, a prospective observational study found no difference in three-month mortality between early and late tracheostomy [3].

Severe acute respiratory syndrome and COVID-19 appeared in Oman in early February 2020, with the number of new cases escalating within days. COVID-19 is a disease that has recently emerged, with many patients requiring mechanical ventilation and ICU admission. During the early period of the COVID-19 pandemic, the guidelines recommended avoiding early tracheostomy to reduce the risk of infection to healthcare providers [4]. Specifically, recommendations for tracheostomy in the pandemic were based on the assumption that maximal infectivity of this novel virus occurred around days 7-10 after symptom onset; thus, performing tracheostomy at that time would expose the medical staff involved to the greatest risk of infection [5].

Several recommendations and guidelines have discussed when to perform tracheostomy in COVID-19 patients, with the timing varying across the literature. Recommendations from the United Kingdom and North America suggested that tracheostomy should be delayed until at least day 14 after symptom onset to allow prognostic information to become clear and for the viral load to decline sufficiently [6]. On the other hand, early tracheostomy may help with early weaning (around 7-10 days) [7]. This study investigates the differences in outcomes when tracheostomy is performed early versus late in COVID-19 and non-COVID-19 cases. Its objectives are to analyze the outcomes of patients with COVID-19 who underwent elective tracheostomy, explore the association between the timing of tracheostomy and the patients' outcomes, and compare them with the results for non-COVID-19 patients. The primary outcome was mortality; the secondary outcomes were ventilation days and ICU LOS.

## Materials and methods

Ethical considerations

This retrospective cohort study was conducted on patients admitted to the ICU at Sultan Qaboos University Hospital between January 2020 and December 2021, including COVID-19 and non-COVID-19 patients. Prior to the study, ethical approval was obtained from the institution's Medical Research Ethics Committee (MREC #2688). The study was then registered with ClinicalTrials.gov (NCT05465837). In accordance with the institutional Medical Research Ethics Committee and the modified Declaration of Helsinki, the privacy of patients was always respected, and their identity was anonymized with codes.

Study participants

Patients >18 years who underwent tracheostomy in the ICU were included in the study, with patients <18 years or those who underwent tracheostomy outside the ICU or those tracheostomized for operative purposes excluded. The 1,393 patients admitted to the ICU during the study were screened, and 156 patients were found to be tracheostomized and included in the study. Age, gender, comorbidities, COVID-19 status, reason for tracheostomy, complications, and ICU course were noted.

Data

Data was obtained from the hospital information system and by reviewing electronic medical records. The data included patient demographic features, previous comorbidities, diagnosis of admission, daily follow-up notes, mechanical ventilation parameters, ventilation days before and after tracheostomy, indication, procedural details, complications, and outcomes (ICU LOS and mortality) of tracheostomy. Tracheostomy was considered to be early when performed within 10 days and late when performed beyond 10 days. Most studies and meta-analyses have used this definition [8].

Bias

This was a retrospective single-center study conducted at a tertiary-level referral center and hence represented a broad segment of the Omani population. The ICU team decided on the time and reason for the tracheostomy, following their protocol based on the latest guidelines, which might have introduced bias into the study.

Study size

This was a time-based study conducted from January 2020 to December 2021, during the COVID-19 pandemic, to compare COVID-19 and non-COVID-19 patients who underwent tracheostomy in the ICU.

Statistical analysis

We used Fisher's exact test to analyze the correlation between categorical variables. To compare the means of independent groups, we used the t-test. The Cochran-Mantel-Haenszel test assessed the conditional independence of categorical predictors associated with categorical outcomes. For the secondary outcomes, the Mann-Whitney U test was used to determine the mean ventilation days after tracheostomy and the LOS in the ICU for each group. For additional analysis, a multivariate binary logistic regression analysis was performed to determine the independent predictors of ICU mortality among the tracheostomized patients for each group. Statistical analysis was performed using IBM SPSS® software, and data projection was performed with STATA 17SE and IBM SPSS.

## Results

**Figure 1:**
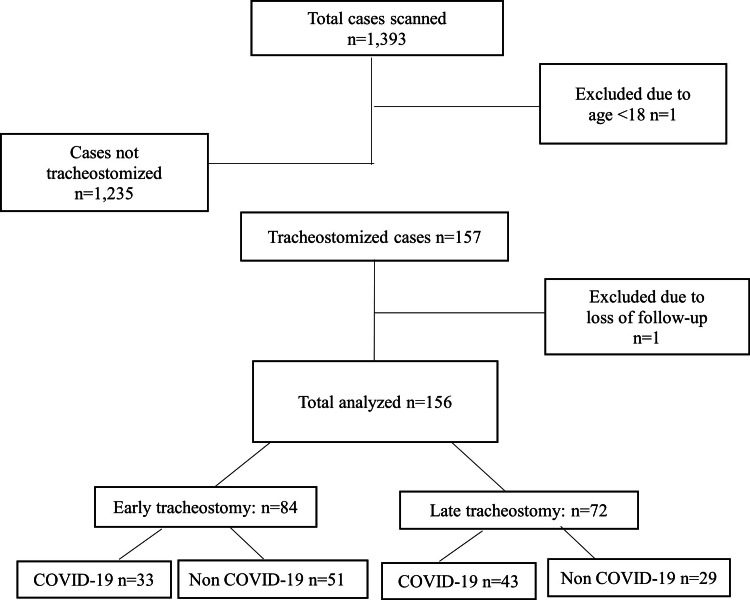
Flow of cases during the study. Early tracheostomy: tracheostomy within 10 days of ventilation; Late tracheostomy: tracheostomy after 10 days of ventilation

All 1,393 patients admitted to the ICU during the study period were scanned, among which 158 patients were found to have undergone tracheostomy. One patient was excluded because he was less than 18. Another patient who was lost to follow-up due to transfer to another hospital was also excluded (Figure 1).

Demographics

Statistical analysis was performed on the 156 tracheostomized patients, of which 102 (65.4%) were males. The mean age was 54.96. The number of patients diagnosed with COVID-19 was 76 (48.7%), and the number of non-COVID-19 patients was 80 (51.3%). Among 156 patients, 114 (73.1%) cases had comorbidities. Hypertension (HTN) was the most common comorbidity (69/156, 44.2%), followed by diabetes mellitus (DM) (66/156, 42.3%). Failure to wean was the most common cause of tracheostomy (91/156, 58.3%), followed by low Glasgow Coma Scale (64/156, 41.0%) and airway issues (1/156, 0.6%). Among the whole group, early tracheostomy was performed for 84 out of 156 (53.8%) patients, and late tracheostomy was performed for 72 out of 156 (46.2%) patients. In the early group, there were 33 COVID-19 cases and 51 non-COVID-19 cases. In the late group, there were 43 COVID-19 cases and 29 non-COVID-19 cases. The demographic and clinical characteristics of the early and late tracheostomy groups are given in Table 1.

**Table 1:** Patient characteristics. SD: standard deviation; DM: diabetes mellitus; HTN: hypertension; IHD: ischemic heart disease; RTA: road traffic accident; ARDS: acute respiratory distress syndrome; GCS: Glasgow Coma Scale

Variable		Early tracheostomy N (%)	Late tracheostomy N (%)
Age (mean (SD))		53 (15.617)	57.25 (15.474)
Gender	Male	54 (64.3)	48 (66.7)
	Female	30 (35.7)	24 (33.3)
Comorbidities	Yes	57 (67.9)	57 (79.2)
	DM	34 (40.5)	32 (44.4)
	HTN	41 (48.8)	28 (38.9)
	IHD	18 (21.4)	12 (16.7)
	Stroke	12 (14.3)	7 (9.7)
	RTA	10 (11.9)	0
	Other neurological conditions	6 (7.1)	7 (9.7)
	ARDS	31 (36.9)	48 (66.7)
COVID-19	N=76 (48.7)	33 (39.3)	43 (59.7)
Non-COVID-19	N=80 (51.3)	51 (63.7)	29 (36.3)
Reason for tracheostomy	Low GCS	45 (53.6)	19 (26.4)
	Failure to wean	38 (45.2)	53 (73.6)
	Airway issues	1 (1.2)	0
Complications	Nil	78 (92.9)	67 (93.1)
	Bleeding	5 (6)	2 (2.8)
	Surgical emphysema	1(1.2)	0
	Pneumothorax	0	1 (1.4)
	Infection	0	2 (2.8)

The patients with COVID-19 infection (P=0.016) and road traffic accident (RTA) (P=0.002) were found to have a significantly higher association with tracheostomy. No complications were documented in 92.2% of cases (145/156). For the remainder, the most common complication was bleeding (7/156, 4.5%). The median follow-up time was 22.5 days (18-32 days), and the maximum was 81 days. There was no missing data for any of the variables for all 156 patients.

Primary outcome

**Figure 2:**
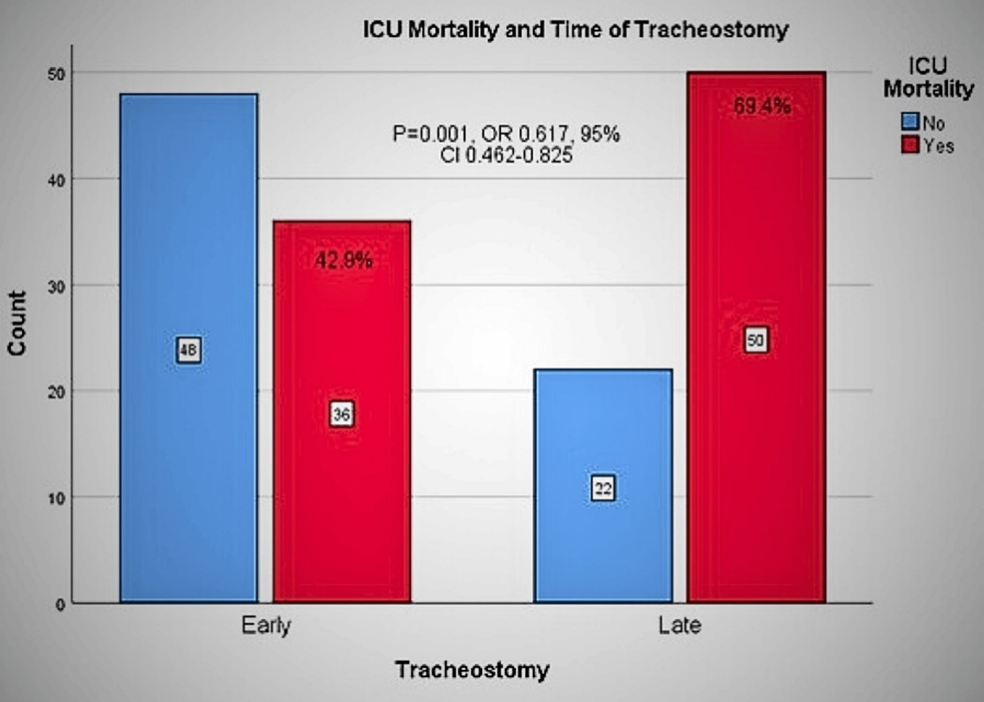
ICU mortality for early versus late tracheostomy groups. Early tracheostomy: within 10 days of ventilation; Late tracheostomy: after 10 days of ventilation; OR: odds ratio; CI: confidence interval; ICU: intensive care unit

The relation between the ICU mortality of tracheostomized patients and the presence and absence of COVID-19 infection was studied. The overall ICU mortality was 55.1% (86/156), with 30-day mortality of 45.5% (71/156) and 60-day mortality of 53.8% (84/156). The mortality was 42.9% (36/84) versus 69.4% (50/72) (P=0.001, OR=3.03, 95% CI=1.563-5.874) in the early versus late group (Figure 2).

**Figure 3:**
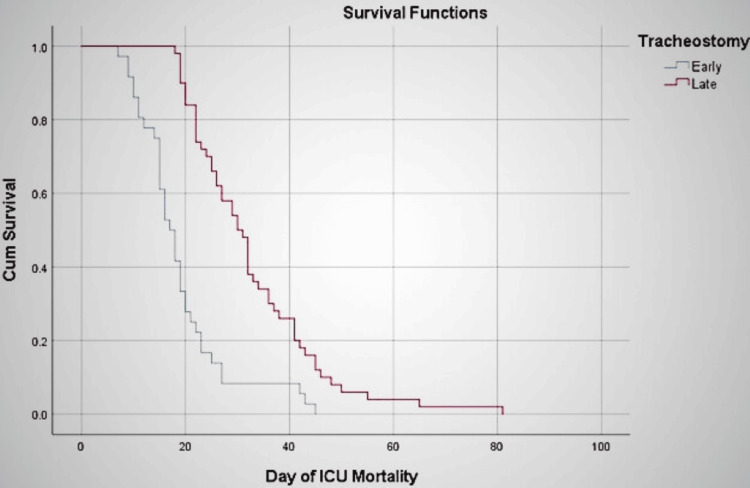
Graph of survival function for early and late tracheostomy groups. P=0.000 (logrank test) Early tracheostomy: within 10 days of ventilation; Late tracheostomy: after 10 days of ventilation; ICU: intensive care unit

The 30-day mortality was higher for the late group but not significantly so. In contrast, the 60-day mortality was significantly higher for the late group (P at 30 days=0.199, P at 60 days=0.04). The absolute risk reduction (ARR) of ICU mortality of early tracheostomy compared with late tracheostomy is 26.5%. The Kaplan-Meier survival analysis reveals a significant difference between the early and late groups in terms of survival (Figure 3).

**Figure 4:**
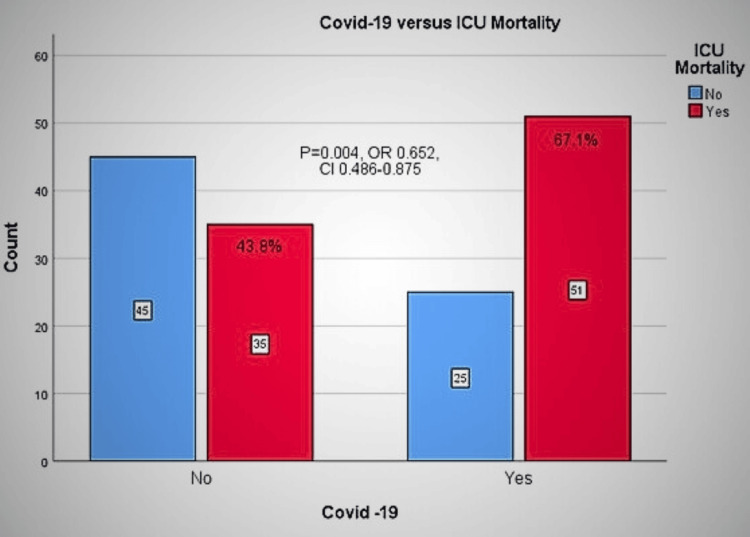
ICU mortality for non-COVID-19 versus COVID-19 cases. OR: odds ratio; CI: confidence interval; ICU: intensive care unit

In the non-COVID-19 group, the overall mortality rate was 43.8%, with a rate of 31.4% in the early group versus 65.5% in the late group. In the COVID-19 group, the overall mortality was 67.1% (60.6% in the early and 72.1% in the late group) (P=0.005, OR=2.640, 95% CI=1.345-5.181) (Figure 4). The ARR of ICU mortality of the non-COVID-19 patients compared with the COVID-19 patients is 23.3%.

Secondary outcomes

For the non-COVID-19 patients, ventilation days after tracheostomy and ICU LOS were significantly lower in the early group (Table 2). For the COVID-19 patients, ventilation days after tracheostomy was similar in both groups. However, ICU LOS was significantly lower in the early group.

**Table 2:** Secondary outcomes. *Mann-Whitney U test ICU: intensive care unit

Non-COVID-19 group	Tracheostomy	Mean (SD)	P-value
Ventilation days after tracheostomy	Early	9.18 (8.099)	<0.001*
	Late	17.10 (11.806)	
ICU length of stay	Early	16.88 (8.128)	<0.001*
	Late	33.72 (12.728)	
COVID-19 group	Tracheostomy	Mean (SD)	P-value
Ventilation days after tracheostomy	Early	14.15 (10.072)	0.821*
	Late	13.86 (9.511)	
ICU length of stay	Early	23.12 (9.908)	0.001*
	Late	30.02 (10.514)	

Multivariate binary logistic regression analysis of the independent predictors of ICU mortality among the tracheostomy patients revealed a significant relation with age, ventilation days before and after tracheostomy, and ICU LOS. This analysis also indicated that the patients with late tracheostomy had double the risk of ICU mortality than those with early tracheostomy after adjusting for the confounding effect of COVID-19 (Table 3).

**Table 3:** Multivariate analysis of factors affecting ICU mortality of tracheostomized patients. CI: confidence interval; DM: diabetes mellitus; HTN: hypertension; IHD: ischemic heart disease; RTA: road traffic accident; ARDS: acute respiratory distress syndrome; ICU: intensive care unit

Variable	B	P-value	Odds ratio	95% CI	
Age	0.030	0.046*	1.031	1.000	1.062
Gender: female	0.118	0.777	1.125	0.497	2.549
DM	0.222	0.643	1.248	0.488	3.189
HTN	0.040	0.937	0.960	0.349	2.640
IHD	0.158	0.774	1.171	0.399	3.435
History of stroke	0.587	0.320	0.556	0.175	1.767
RTA	0.150	0.875	1.162	0.177	7.626
COVID-19	0.056	0.952	1.058	0.168	6.675
ARDS	0.839	0.382	2.314	0.353	15.16
Tracheostomy: late	0.810	0.183	2.248	0.682	7.415
Type of tracheostomy: percutaneous	0.328	0.606	0.720	0.207	2.506
Ventilation days before tracheostomy	0.239	0.020*	1.270	1.039	1.552
Ventilation days after tracheostomy	0.266	0.005*	1.305	1.084	1.571
ICU length of stay	0.249	0.008*	0.780	0.649	0.937

## Discussion

This study found that the mortality rate was higher for late tracheostomy in both the COVID-19 and non-COVID-19 groups, with the risk of mortality twice as high for the late group as for the early group. The patients with late tracheostomy in the non-COVID-19 group had more ventilation days after tracheostomy as compared to early. Ventilation days after tracheostomy were almost the same for early and late tracheostomy in the COVID-19 group. ICU LOS was longer for the late group than for the early group in both the COVID-19 and non-COVID-19 groups. The overall ICU mortality was higher in the late group than in the early group. The additional analysis revealed a significant association between ICU mortality, age, ventilation days before and after tracheostomy, and ICU LOS.

Multiple studies have compared the outcomes of early versus late tracheotomy on critically ill patients undergoing mechanical ventilation [2,9-14]. The proper timing of tracheostomy for intubated patients is still under debate. A recent meta-analysis by Battaglini et al. on tracheostomized COVID-19 patients concluded that timing and tracheostomy technique did not affect the outcomes, including mortality and ICU stay [11]. Another study by Blot et al. found no significant difference in mortality, ICU stay, degree of sedation, and complications between early and late tracheostomy among general ICU patients [12].

On the other hand, several RCTs, meta-analyses, and observational studies reported a better outcome with early tracheostomy than with late tracheostomy. A recent meta-analysis by Deng et al. found that early tracheostomy in COVID-19 patients can reduce ICU LOS and mechanical ventilation duration [13]. In a meta-analysis by Andriolo et al., seven out of eight RCTs found lower mortality rates for early tracheostomy than for late tracheostomy [2]. An observational study by Livneh et al. concluded that decannulation rates were significantly higher and mortality rates were non-significantly lower in the early tracheostomy group [14]. The optimal time of early tracheostomy is still under investigation. A recent meta-analysis by Chorath et al. found that tracheostomy within seven days of mechanical ventilation (instead of 10 days) is associated with reduced ventilator-associated pneumonia, fewer ICU days, and more ventilator-free days [9]. On the other hand, the TracMan randomized trial indicated no association between tracheostomy within four days of critical care admission and an improvement in 30-day mortality or other important secondary outcomes [15]. Our study adds an important insight into the timing for COVID-19 and non-COVID-19 cases and agrees with the studies favoring early tracheostomies for worthy patients.

Strengths

One of the strengths of this study is its large sample size. In addition, it is one of the few studies examining the impact of the timing of tracheostomy on a population that includes both COVID-19 and non-COVID-19 patients.

Limitations

One of the limitations of this study is that it is a retrospective study performed at a single tertiary center. In addition, the timing of the tracheostomy was decided by the treating intensivist consultant, although standard protocols were followed. Another limitation is the evolution of different management strategies for COVID-19 infection during the pandemic. For example, the recommendation of starting dexamethasone for all patients requiring oxygen support by recovery trial was implemented halfway through the pandemic. The proportion of patients tracheostomized before and after such novel treatment strategies could differ. In addition, the mortality rates for the COVID-19 groups with early and late tracheostomy could have been higher because of the nature of the disease itself.

## Conclusions

This study found that early tracheostomy is associated with better outcomes than late tracheostomy in terms of mortality in both COVID-19 and non-COVID-19 groups. In addition, the secondary outcomes of this study also show that when tracheostomy is performed within 10 days of intubation, there are fewer ventilation days and shorter ICU stays for both COVID-19 and non-COVID-19 groups. Hence, early tracheostomy is found to be beneficial regardless of the presence of COVID-19 infection in this study.
